# Modular vaccine platform based on the norovirus-like particle

**DOI:** 10.1186/s12951-021-00772-0

**Published:** 2021-01-19

**Authors:** Vili Lampinen, Suvi Heinimäki, Olli H. Laitinen, Marko Pesu, Minna M. Hankaniemi, Vesna Blazevic, Vesa P. Hytönen

**Affiliations:** 1 Faculty of Medicine and Health Technology, Tampere University, 33014 Tampere, Finland; 2 Vaccine Development and Immunology/Vaccine Research Center, Faculty of Medicine and Health Technology, Tampere University, Tampere, Finland; 3 Fimlab Laboratories, Tampere, Finland

**Keywords:** VLP, Vaccine, Influenza, SpyCatcher, Virus

## Abstract

**Background:**

Virus-like particle (VLP) vaccines have recently emerged as a safe and effective alternative to conventional vaccine technologies. The strong immunogenic effects of VLPs can be harnessed for making vaccines against any pathogen by decorating VLPs with antigens from the pathogen. Producing the antigenic pathogen fragments and the VLP platform separately makes vaccine development rapid and convenient. Here we decorated the norovirus-like particle with two conserved influenza antigens and tested for the immunogenicity of the vaccine candidates in BALB/c mice.

**Results:**

SpyTagged noro-VLP was expressed with high efficiency in insect cells and purified using industrially scalable methods. Like the native noro-VLP, SpyTagged noro-VLP is stable for months when refrigerated in a physiological buffer. The conserved influenza antigens were produced separately as SpyCatcher fusions in *E. coli* before covalent conjugation on the surface of noro-VLP. The noro-VLP had a high adjuvant effect, inducing high titers of antibody production against the antigens presented on its surface.

**Conclusions:**

The modular noro-VLP vaccine platform presented here offers a rapid, convenient and safe method to present various soluble protein antigens to the immune system for vaccination and antibody production purposes.
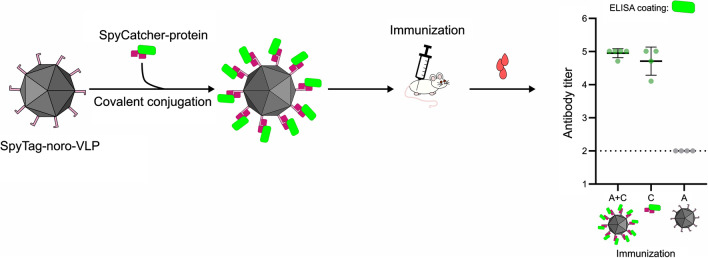

## Background

Out of all medical inventions, vaccination has doubtless had the largest impact on global health, but this mature invention still has potential for improvement. A well-known example of this is the influenza vaccine, which because of the rapid evolution of influenza virus surface proteins, needs reformulation for each season [1]. Influenza has imposed a considerable disease burden worldwide for hundreds of years and it still constitutes a constant threat to public health. On a global scale, WHO estimates the yearly death toll of influenza infections between 290,000 and 650,000 [2], while emerging pandemic strains threaten the lives of millions. To avoid the current need of annual vaccination, we need a universal influenza vaccine that targets conserved regions that mutate at a slower pace in the virus. Most influenza vaccines in use today are still produced as whole virus vaccines in chicken eggs, using the same technology that has been in use since 1946 [3]. This suffices against the particular strain chosen for production, but more modern approaches are needed for rapid and dependable production of vaccines that work against multiple strains of influenza.

Virus-like particles (VLPs) have recently shown great promise as flexible, safe and effective modern vaccines. They are virus-genome-free particles that are similar in size and shape to the respective viruses. This makes VLPs incapable of infection but still very effective in mounting immune responses [4]. VLPs are produced by recombinant expression of structural virus proteins, followed by their spontaneous assembly into particles. In addition to the elementary idea of replacing inactivated virus vaccines with their safer and more producible VLP counterparts, VLP technology can be harnessed against any pathogen by decorating VLPs with antigens from heterologous pathogens. The simplest way to do this is to genetically fuse the antigens to a virus capsid protein that participates in forming the VLP. However, as this often hinders either VLP or antigen assembly, it requires laborious and time-intensive planning and optimization individually for each antigen tested [5]. Using a modular system, wherein the antigen and VLP are produced separately and conjugated together only after that, circumvents these problems. Both native and decorated VLP vaccines, such as vaccines against human papilloma virus and malaria [Gardasil (MSD, Ireland), and Mosquirix H-W-2300 (GSK, UK), respectively], are already in clinical use, which demonstrates the commercial feasibility and medical potential of VLP technology. The modular VLP vaccine approach is not yet in clinical use, but with the research reported here, we aim to append to previous research on the subject and bring it closer to clinical testing.

We have previously demonstrated that the norovirus-like particle (noro-VLP) shows exceptional producibility and stability, even when displaying C-terminal HisTags on its surface for simple non-covalent and modular decoration [6]. Now, we developed this idea further by displaying SpyTags on the noro-VLP and covalently decorating it with SpyCatcher conjugation technology [7]. With this modular system, we can produce and purify SpyCatcher-fused antigen separately from the SpyTag-noro-VLP and then decorate the noro-VLP via isopeptide bonds forming spontaneously between SpyTag and SpyCatcher. To test a medically relevant application of this flexible molecular platform, we produced two conserved influenza antigens as SpyCatcher fusions to present them on the noro-VLP. The noro-VLP was decorated with the ectodomain of influenza matrix-2 ion channel protein (M2e) and a minimized stem-fragment of hemagglutinin glycoprotein 2 (HA2), and the immunogenicity of the decorated particles was tested in BALB/c mice. Both protein fragments are highly conserved across different influenza strains from a long time span [8]. A vaccine that can create an immune response against these conserved influenza protein sequences could protect against many pathogenic influenza strains without annual renewal [9, 10]. Generating such a response has proven near impossible when vaccinating with or getting infected by natural viruses, presumably because both conserved regions are immunogenically dominated by the highly variable regions in the highly immunogenic and prominent head domains of HA and neuraminidase (NA) proteins. When fused to a VLP, only the desired conserved antigen fragments are displayed in an immunogenic way. The M2e peptide and the trimeric HA2 protein domain are disparate antigens physically, so successful conjugation of them both on the noro-VLP holds great promise for wide use of the noro-VLP platform in biotechnology.

## Results

### Production of SpyTagged norovirus-like particles and SpyCatcher fusion proteins

**Fig. 1:**
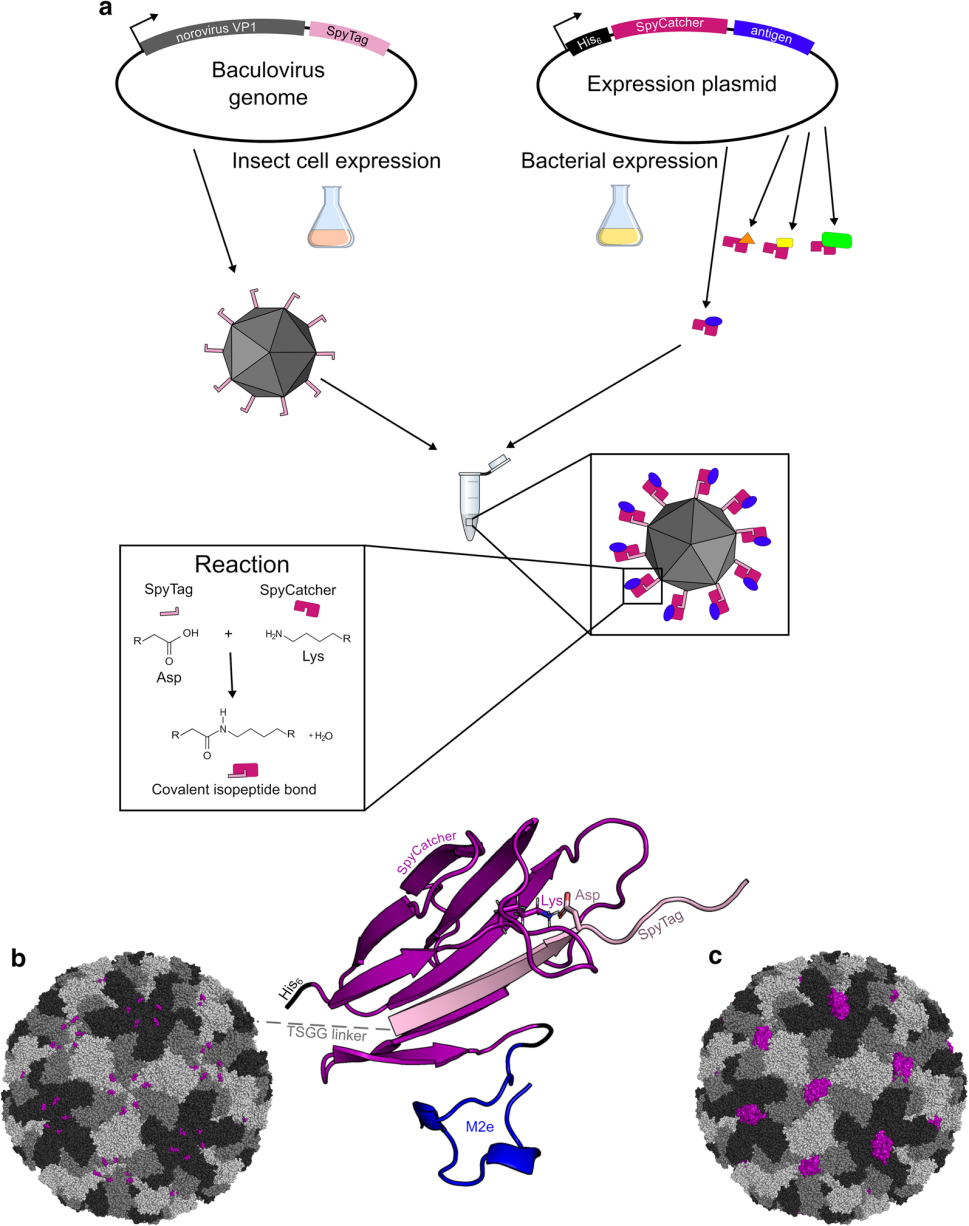
Overview of SpyTag-norovirus-like particle vaccine design. **a** When SpyTagged norovirus VP1 protein is expressed in insect cells, the proteins spontaneously assemble to form noro-VLPs with SpyTag peptides protruding from their surface. Then, this SpyTag-norovirus-like particle is mixed with a separately produced SpyCatcher-antigen fusion protein (depicted here as blue/orange/yellow/green antigen on purple SpyCatcher), and a biologically irreversible covalent bond forms in between. **b** The noro-VLP is formed by 180 VP1 proteins, whose C-termini (purple) protrude from the surface in clusters of five or six. SpyTag (light purple) was genetically fused to the C-terminus of noro-VP1 via a 4-residue linker. On a different scale, the panel shows a conjugated SpyCatcher (dark purple), fused to M2e (blue) by its C-terminal. **c** One SpyCatcher bound per cluster of SpyTags is shown for size comparison. The fusion protein models in **b** and **c** were constructed with the SynLinker web application [46] (maintenance ceased since) and visualized with PyMOL [47]. The models are based on the following RCSB PDB structures: norovirus: 1IHM, SpyCatcher: 4MLS, M2e: 4N8C

**Fig. 2:**
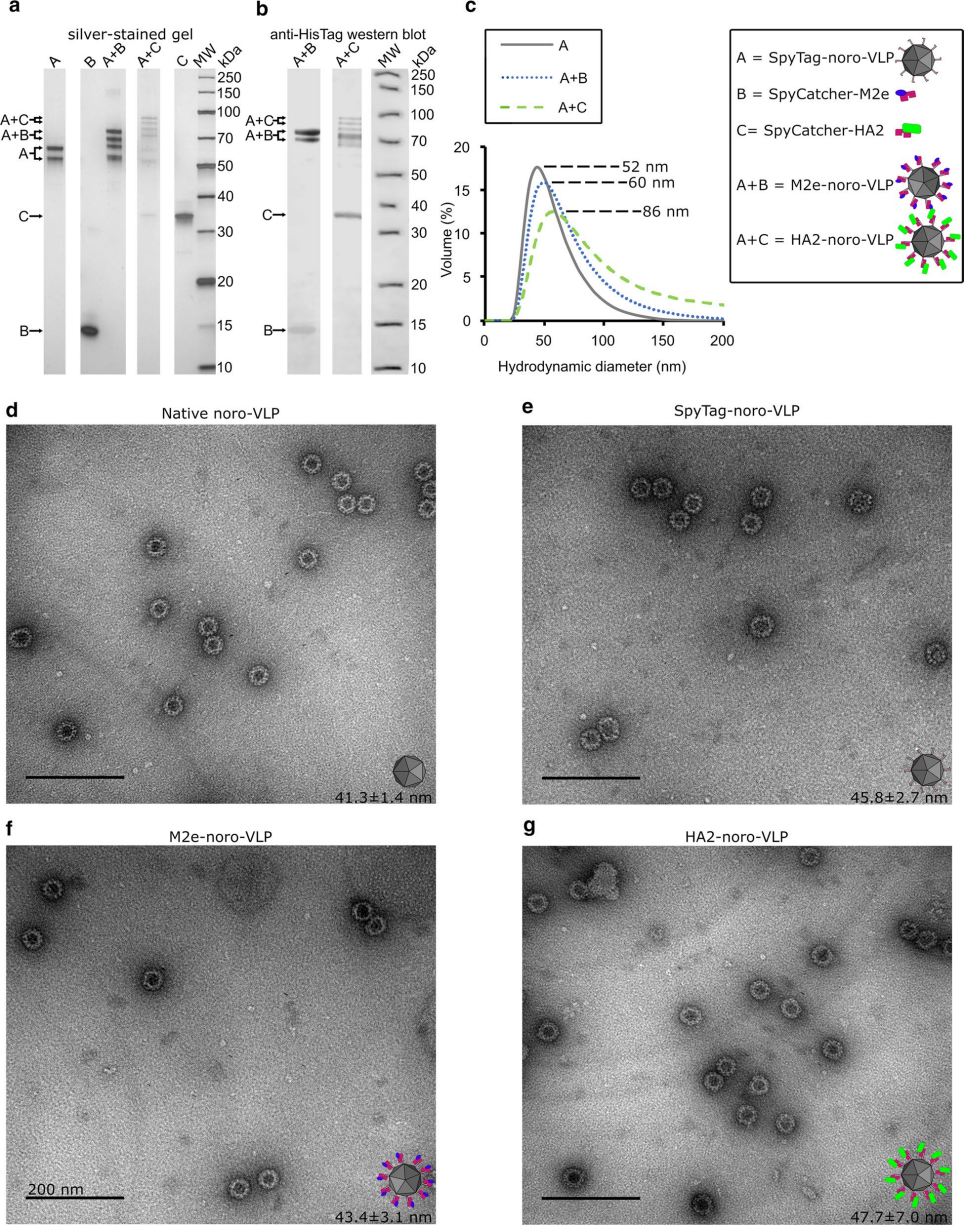
Conjugation of norovirus-like particles. **a** Silver-stained SDS-PAGE gel of the vaccine components. The noro-VLP appears as a double band between the 70 and 50 kDa markers. All wells contain approximately 250 ng of protein. **b** Western blot analysis of noro-VLP decorated with the influenza antigens. The HisTag in the N-termini of SpyCatcher fusion proteins is recognized by mouse anti-HisTag antibody. **c** Dynamic light scattering analysis of SpyTag-noro-VLP and SpyTag-noro-VLP decorated with M2e and HA2. Numerical data shown in Additional file 1: Figure S4. Representative transmission electron microscopy pictures with arithmetic means of equivalent circle diameters ± standard deviation of **d** native noro-VLP (n = 114), **e** SpyTag-noro-VLP (n = 112) and noro-VLP decorated with **f** M2e (n = 109) and **g** HA2 (n = 274). The scale bars are all 200 nm. 60,000× magnification was used for these pictures and analysis

To create norovirus-like particles decorated with influenza antigens, we expressed and purified SpyTagged norovirus-like particles (SpyTag-noro-VLPs) separately from SpyCatcher-fused antigens before conjugating the components together (overview in Fig. 1). The best SpyTag-noro-VLP yields were obtained by infecting Hi5 insect cells with SpyTag-noro-VLP-expressing baculovirus at a multiplicity of infection (MOI) value of 1 and proceeding to the first crude purification step directly after collecting the expressed protein. Both tangential flow filtration (TFF) and ultracentrifugation through a sucrose gradient separated most protein impurities from the VLP (data not shown). After this crude purification, SpyTag-noro-VLPs could be stored at + 4 °C for ≥ 5 months without apparent change in morphology, homogeneity, concentration or conjugation efficiency of the product. However, a polishing ion exchange step is required to separate the similarly sized baculovirus from the SpyTag-noro-VLP [11]. After anion exchange chromatography purification, the SpyTag-noro-VLP yields were 10–30 mg/L of insect cell culture, with a purity of > 95% (Fig. 2a), as determined by densitometry analysis from silver-stained SDS-PAGE gel. Anion exchange chromatography removed all detectable baculovirus from the product (determined by anti-gp64 western blotting, Additional file 1: Figure S1), making it suitable for vaccination purposes.

We expressed influenza M2e and HA2 as SpyCatcher-fusion proteins in *E. coli* and purified them with Ni–NTA affinity chromatography. For M2e, we chose the human influenza virus consensus sequence of the first 24 N-terminal amino acids of influenza matrix-2 [10], while HA2 here is the minimized, pre-fusion stem region of influenza hemagglutinin, first reported in [12]. Like the original authors, we included a foldon trimerization domain to the C-terminal of HA2 to support its natural trimerization. A proteolytically cleavable HisTag was included in the N-terminus of SpyCatcher in both fusion proteins to allow purification and detection. The amino acid sequences of the proteins produced in this study are shown in Additional File 1: Table S1. The single-step affinity chromatography purification yielded > 30 mg/L of SpyCatcher-M2e and > 10 mg/L of SpyCatcher-H1F. Both antigens were > 95% pure (by SDS-PAGE densitometry analysis, Fig. 2a) after total protein staining. By including a detergent washing step into the affinity chromatography protocol, we removed most endotoxins from these *E. coli-*expressed proteins to make them usable as vaccine components.

According to dynamic light scattering (DLS) analysis, SpyTag-noro-VLP and SpyCatcher-H1F were very monodisperse (polydispersity index (PdI) < 0.2) and soluble in PBS after purification. SpyCatcher-M2e showed a PdI of over 0.8 in DLS, which would indicate it being a very polydisperse sample, but on the other hand, visual inspection showed no aggregates even in our high concentration samples. In transmission electron microscopy analysis, the produced SpyTag-noro-VLPs showed a similar size and morphology as compared to native noro-VLPs (Fig. 2d, e). MALDI-MS analysis confirmed that SpyCatcher-M2e and SpyCatcher-H1F were intact and had the predicted masses of 14 and 31 kDa, respectively (Additional file 1: Figure S2).

### Decoration of norovirus-like particles with SpyCatcher-fused antigens

The SpyTag-noro-VLPs were decorated with SpyCatcher-fused influenza antigens by mixing the components together in PBS buffer, as described in [13]. The conjugation efficiency was estimated from SDS-PAGE gel by densitometry. Decoration with SpyCatcher-M2e or SpyCatcher-H1F moves the noro-VLP-specific double band upwards by 14 or 31 kDa, respectively (Fig. 2a). This matches the sizes of the SpyCatcher fusion proteins. In both cases, approximately half of the VP1 proteins in the product are covalently conjugated with antigen. Since noro-VLP consists of 180 VP1 proteins [14], 50% conjugation efficiency equals 90 SpyCatcher-antigen molecules per particle. By performing multiple independent conjugation reactions, we noted that the reaction proceeds to the end in 2 h at RT or overnight at + 4 °C. The HisTag in the N-terminus of SpyCatcher-fusion protein is visualized in conjugated VLP bands in anti-HisTag western blot, whereas unconjugated VLP is invisible in these wells (Fig. 2b). All detectable unbound SpyCatcher-M2e is separated in dialysis, but some noncovalently bound SpyCatcher-HA2 can be seen in the gel even after dialysis. Moreover, an extra double band can be seen in the HA2-noro-VLP well between the expected conjugation bands and noro-VLP bands.

In TEM, no obvious differences between native, tagged or decorated particles were initially recognized (Fig. 2d–g), but a particle size distribution measurement from TEM images revealed a measurable increase in particle size in HA2-decorated particles. DLS shows a 15% increase in hydrodynamic diameter when SpyTag-noro-VLPs are decorated with SpyCatcher-M2e and a 65% increase with SpyCatcher-HA2 (Fig. 2c). The low molecular weight of SpyCatcher-M2e could explain why it is not detected in TEM. Decorating the particles also increases the polydispersity index from 0.107 to 0.167 in DLS, but volume distribution still shows only a single, slightly wider peak. Even though the SpyCatcher-antigen fusion proteins were produced in *E. coli*, Triton X-114 washing of the produced proteins reduced the endotoxin content to acceptable levels [15]. All immunogens used in vaccination had an endotoxin content of < 0.054 EU/µg (Table 1) and < 0.18 ng dsDNA/µg of protein.

**Table 1:** Vaccine groups and doses used in the immunization experiment

Group	Mice/group (n)	Vaccine	Total protein dose (µg)	Antigen^a^ dose (µg)	Endotoxin dose (EU)
I	4	SpyTag-noro-VLP^b^	25	24.3	< 1
II	4	SpyCatcher-HA2	25	12.5	< 0.4
III	5	HA2-noro-VLP	15	1.3	< 0.6
IV	4	SpyCatcher-M2e	100	24.3	< 5.4
V	5	M2e-noro-VLP	50	1.2	< 0.4

^a^The dose of the underlined antigen molecule is estimated in this column, as described in the text

^b^Norovirus-like particle

### SpyTagged noro-VLPs are exceptionally stable and convenient to store

**Fig. 3:**
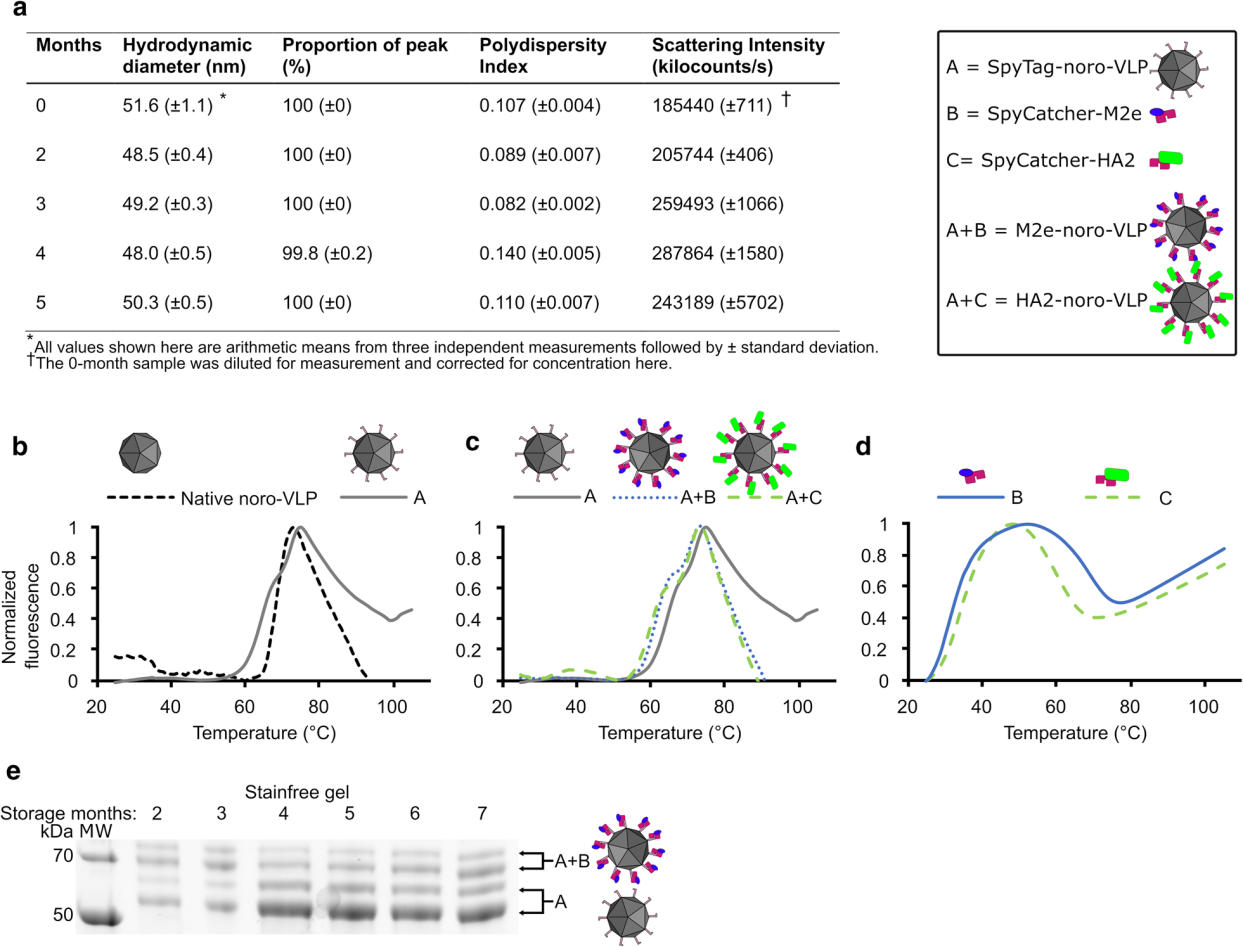
SpyTagged noro-VLPs show high stability and storability. In these stability experiments, the noro-VLPs were stored in PBS (137 mM NaCl, 2.7 mM KCl, 8 mM Na2HPO4, 2 mM KH2PO4, pH 7.4. **a** Dynamic light scattering analyses executed monthly during a 5-month storage period at + 4 °C. Graphical data shown in Additional file 1: Figure S4. **b**–**d** The melting temperatures (Tm) of native noro-VLP (**b**), SpyTag-noro-VLP (**b**, **c**), M2e- and HA2-conjugated SpyTag-noro-VLP (**c**), SpyCatcher-M2e (**d**) and SpyCatcher-HA2 (**d**) were measured with differential scanning fluorimetry (DSF). The fluorophore, SYPRO Orange, binds hydrophobic areas that emerge from the particles and proteins as they unfold upon heating from 25 to 105 °C. Water quenches the fluorescence of SYPRO Orange, so fluorescence increases as more protein becomes unfolded and thus available for binding. T_m_ was calculated from the midpoint of each transition peak. Plotted here are the arithmetic means of normalized fluorescence from three independent measurements. **e** SpyTag-noro-VLP aliquots were stored at + 4 °C and conjugated with SpyCatcher-M2e monthly. The SpyCatcher-M2e aliquots used for this were stored at − 20 °C until conjugation. After overnight conjugation, the reaction was stopped by boiling in SDS

Like the native noro-VLP and norovirus itself, also SpyTag-noro-VLP shows remarkable stability. Due to the influence of temperature on vaccine stability, the stability of the vaccines was evaluated by differential scanning fluorimetry (DSF). DSF measurements showed that SpyTag-noro-VLP’s melting temperature (T_m_) of + 66(± 0.1) °C in pH 7.4 PBS is very close to that of native noro-VLP’s + 68(± 0.1) °C (Fig. 3b). Our comparison of the native and SpyTagged noro-VLP under pH 3, 5.5 and 8 (Additional file 1: Figure S5) confirmed that SpyTag destabilizes the noro-VLP only in the slightest and that noro-VLP is most stable at mildly acidic to neutral pH. This conforms with earlier studies on noro-VLP stability [16]. Conjugating noro-VLP with M2e or HA2 lowered its T_m_ further to + 63(± 0.1) °C (Fig. 3c). HA2-noro-VLP shows signs of a second unfolding peak near SpyCatcher-HA2′s own T_m_ of + 35(± 0.1) °C.

In series of DLS measurements, a SpyTag-noro-VLP sample did not show any signs of aggregation or disintegration during a five-month follow-up (Fig. 3a). During the study, we stored the purified SpyTag-noro-VLP in PBS at + 4 °C. In seven months, we did not observe a change in conjugation efficiency (Fig. 3e). For the stability experiment, SpyCatcher-M2e aliquots were stored at − 20 °C and thawed each month before the conjugation reaction.

Although the vaccine components are easy to store on their own, we noticed that after conjugation, HA2-conjugated noro-VLP lost some of its stability. During storage of conjugated HA2-noro-VLP vaccine sample, the largest bands on SDS-PAGE disappeared after 76 days at + 4 °C (Additional file 1: Figure S3). At the same time, prominence of a 13-kDa band, unrecognized by anti-HisTag antibodies, increased. Addition of 1 µg/mL aprotinin and leupeptin protease inhibitors into a parallel sample prevented these changes. By 40 days, the changes in SDS-PAGE appearance were not yet apparent, meaning that the phenomenon should not influence the immunizations. It should be noted that these observations were made with samples wherein the unreacted H1F was still unseparated from the mixture.

### Decorated norovirus-like particles induce strong antibody responses

**Fig. 4:**
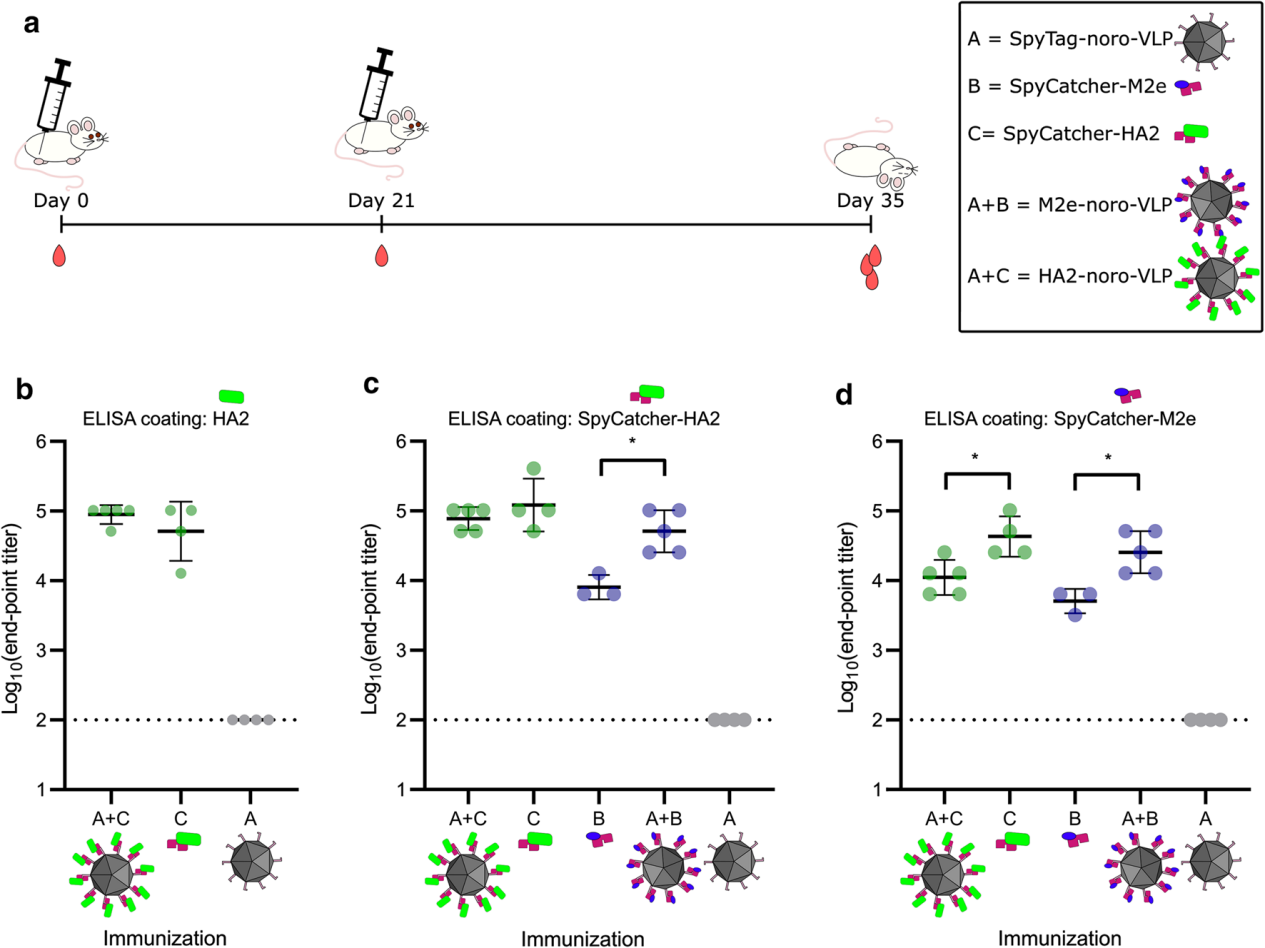
The vaccine components induce high titers of antibodies in BALB/c mice. **a** A schematic showing the timeline of the vaccinations in BALB/c mice. We tested the immunogenicity of the vaccine candidates by injecting 50 µL of each non-adjuvanted compound i.m. to four or five mice, according to Table 1. **b**–**d** Log10 transformations of IgG antibody titers against HA2 (**b**), SpyCatcher-HA2 (**c**) or SpyCatcher-M2e (**d**), as measured in ELISA wells coated with the recombinant protein indicated above each graph. Mean titers are represented by the thick line ± standard deviation; * symbolizes significant difference with p < 0.05, determined by Mann–Whitney U-test. Each dot represents a single mouse. Undetectable antibody levels were denoted with the titer 100 (dashed line)

To study the immunogenicity of the purified and decorated noro-VLPs, we used them to immunize female BALB/c mice intramuscularly. Groups of 4–5 mice were injected twice with M2e or HA2 decorated noro-VLPs or with control samples, with a 3-week interval. We used ELISA to analyze the IgG antibody responses in mice sera. Mice vaccinated with SpyCatcher-HA2 produced high titers (> 10^4^) of HA2-specific antibodies, regardless of whether HA2 was presented on noro-VLP or introduced alone as a SpyCatcher fusion protein (Fig. 4b). The mean titer was slightly higher in the HA2-noro-VLP group (p = 0.40) and noro-VLP presentation of HA2 decreased the deviation in the immune response between mice by 68%. Presenting the M2e peptide on noro-VLP via SpyCatcher linkage produced no detectable M2e-specific antibodies (not shown). Instead, the antibody titers seen in Fig. 4c are more likely to recognize SpyCatcher.

By cross testing our SpyCatcher fusion proteins, we can estimate the titer of antibodies formed against the common factor of the vaccine components—SpyCatcher. Antibodies against SpyCatcher were produced, but it is not clear if noro-VLP presentation enhanced their formation or not. SpyCatcher-M2e on noro-VLP induced more SpyCatcher-antibodies than SpyCatcher-M2e alone (p = 0.036) (Fig. 4c), but conversely, SpyCatcher-HA2 raised more antibodies against SpyCatcher than HA2-noro-VLP (p = 0.032) (Fig. 4d).

## Discussion

While COVID-19 has recently eclipsed seasonal influenza as a viral threat, it still serves as a shocking reminder of the insurance that functional virus vaccines provide us. Although it has been more than 70 years since the first influenza vaccine, most vaccines are still made with the same egg-based manufacture technology. The procedure is simple and usually functional, but also poses several problems, including egg allergy-related side effects, limitations in egg availability and requiring work with live, infectious viruses [3]. Even though influenza is used as a well-known example here, credible alternative vaccine technologies to any whole pathogen vaccine have arrived only recently in the form of DNA, RNA and recombinant protein vaccines. While all nucleic acid vaccines for influenza remain exclusively in research use, the first recombinant influenza vaccine called Flublok (Sanofi Pasteur, Lyon, France) was approved for commercial use in the USA in 2013. Flublok is comprised of recombinant full-length, wild-type HA protein. It does not provide any broader immunity than conventional, egg-based influenza vaccines, but avoids problems related to virus safety and egg allergies and availability. Additionally, generation of a new vaccine product with recombinant technology can be significantly faster [17]. Most recombinant proteins, including influenza HA in Flublok, are too small to be very immunogenic without addition of strong adjuvants [18]. Recombinantly produced VLPs, on the other hand, assemble into a virus-sized particle that can drain into lymph nodes and cross-link B-cell receptors, generating strong immune responses even without added adjuvants [4].

The recently developed SpyCatcher/SpyTag conjugation has been used before for presenting SpyTagged malaria antigens on SpyCatcher-decorated bacteriophage AP205 VLPs [13]. In the present study, we adapted the SpyCatcher/SpyTag technology for decorating the exceptionally stable norovirus-like particle. The methods described here can be used to establish large-scale production of vaccine-grade SpyTag-noro-VLPs and SpyCatcher fusion proteins. Production of two SpyCatcher-fused influenza antigens succeeded in high efficiency in a simple *E. coli* batch production system. This was expected, as SpyCatcher has been shown to be a tenaciously folding protein that can even enhance the solubility of its fusion partners [19]. Arrays of SpyCatcher fusion proteins have been successfully produced in different systems after the publication of the technology (e.g. [19–21]). SpyCatcher functions as N- or C-terminal or even internal loop fusions [7], which makes our vaccine platform compatible with the vast majority of soluble protein antigens. The SpyTag-noro-VLP was decorated with SpyCatcher fusion proteins by simply mixing the components together in solution. In this study, we used PBS, in which noro-VLP remains stable for months (Fig. 3 and [22]), but SpyCatcher works in a range of different buffers and conditions [7]. Generating each new vaccine in an identical way as SpyCatcher fusions presented on noro-VLP would expedite the regulatory process compared to a different vaccine formula for each generation or kind of pathogen.

Efficient and scalable production is imperative for a functional vaccine platform, since it needs to be manufactured quickly in enormous quantities against emerging epidemics and pandemics. The norovirus-like particle production methods presented here and before [23] do not require specialized virus-outbreak-safe facilities and they are fully scalable even to industrial level with standard cell culture and protein purification equipment. To exemplify, it has been estimated that to achieve global herd immunity against the current COVID-19 pandemic, close to 5 billion vaccine doses are needed. Given a typical VLP vaccine dose of 120 µg (e.g. Gardasil, MSD, Ireland), this would mean a whopping 24 million liters of insect cell culture, even with no boosts and a yield of 10–30 mg. Although this sounds like a large volume and an immense effort, it is still vastly more realistic than the 4–8 billion infected chicken eggs it would take to produce this number of vaccine doses the traditional way [24].

Decoration of noro-VLPs worked well with methods described previously [7, 13, 19] with a simple M2e peptide and a complicated, trimeric HA2 protein. With noro-VLP, the conjugation efficiency was estimated to be up to 50%, instead of close to 100% that has been reported with other SpyCatcher-VLP systems [13, 19]. Based on the X-ray structure of noro-VLP (Fig. 1, panels B-C), we predicted that up to three SpyCatcher molecules can fit into one SpyTag cluster on the SpyTag-noro-VLP. The clusters contain five or six C-termini, hence five or six SpyTags, which would translate into a maximum conjugation efficiency of ~ 60%. This equals 108 antigen molecules per particle, which is in line with similar VLP platforms [19, 25, 26].

We used SDS-PAGE to measure the mobility shift caused by conjugation of the vaccine components and densitometry to compare the ratios of unconjugated and conjugated noro-VLP bands. In SDS-PAGE, covalently bound peptides (e.g. proteins conjugated via SpyCatcher/SpyTag) are expected to form a single band and noncovalently bound peptide complexes (e.g. the 180 proteins that form the noro-VLP together) to separate into their peptide components. In SDS-PAGE gels, the noro-VLP double band moved upwards by the size of its SpyCatcher-antigen partner (Fig. 2a). The noro-VLP double band is caused by an N-terminal truncation [27], which means that both the full length and truncated forms have SpyTag in their C-termini. Both forms participate in forming the particle. M2e conjugation behaved as expected on gel. However, HA2-noro-VLP shows up as the expected double band at 92/89 kDa, but also as a double band near 75/72 kDa. All four conjugation bands are recognized by anti-HisTag antibody (Fig. 2b), which indicates that SpyCatcher’s N-terminal HisTag must be included in these forms as well. We hypothesize that partial cleavage takes place after conjugation of the trimeric HA2-SpyCatcher on SpyTag-noro-VLP, cutting loose some of covalently conjugated HA2 and leaving only SpyCatcher on some binding spots on the noro-VLP. On the other hand, we also observed that some SpyCatcher-HA2 remains in the vaccine product after vigorous dialysis, so some of the protein may bind the large noro-VLP only through noncovalent means via its foldon trimerization domain. Based on the immunization results, enough HA2 remained bound on the noro-VLP for producing anti-HA2 antibodies.

Earlier research shows that native noro-VLPs are exceptionally stable and easy to store [22], which probably derives from the known perseverance of the pathogenic norovirus. This study confirms that the same aspects apply to the SpyTag-noro-VLP. The SpyTag-noro-VLP can be stored at + 4 °C for months in a simple PBS buffer without any additives. This is important for making large stockpiles of the vaccine platform to secure supply in an emergency. For example, in developing countries, continuous refrigeration is not always possible, so storage of noro-VLP at room temperature should still be investigated. Since the native noro-VLP can already survive 7 days at room temperature [22], noro-VLP is a promising platform for stabilization studies. For example, stabilization via added disulfide bonds and formalin treatment has increased the thermostability of many VLPs before [28]. Formalin treatment of Coxsackievirus VLPs improved both their stability and immunogenicity [29], which demonstrates the importance stability can have on the clinical applicability of vaccines.

As a downside to this vaccine platform, SpyCatcher fusion proteins are not as stable as the noro-VLP, according to their melting temperatures (Fig. 3d), so we stored these in frozen form. Fusing the M2e peptide or HA2 on SpyCatcher apparently lowered its melting temperature from + 49 °C [30] to + 31 or + 35 °C, respectively. The effect of these fusions on the observed melting temperature of SpyCatcher seems stronger than fusion of beta lactamase [31]. Also, we noticed degradation of conjugated HA2-noro-VLP during its storage at + 4 °C after 76 days. A minor protease contamination, most likely from *E. coli*, could explain this observation. It should still be investigated if further purification steps could resolve the issue altogether. On the other hand, the process seems to be visible only after over a month of storage, so it is possible to still use antigens like described above if administration is done within a month after conjugation. The dialysis step would be impossible to do in the clinic, but SpyCatcher conjugation should proceed to the end within minutes also with equimolar concentrations, especially with more optimized SpyCatcher versions [7, 21, 30]. Conjugation could be done in situ for example by mixing of two pre-prepared vaccine component solutions with a dual chamber syringe.

In the ongoing effort to make a universal influenza vaccine, recombinant vaccines containing conserved peptides and protein domains from M2 and HA have been studied extensively. HA is natively formed by six non-covalently bound HA1 and HA2 polypeptides, so recombinant expression of the conserved stem domain is not trivial. The problem was solved by engineering a construct that fused the structurally and immunologically important stem fragments of HA1 into the gene of HA2 to make a single soluble polypeptide, capable of forming a trimer that mimics the native stem domain of HA [12]. The authors immunized mice with this HA ministem based on H1 influenza, together with the respective antigen from an H5 influenza strain. The vaccine protected mice from influenza strains H1, H3 and H5, which was the broadest protective effect reported thus far [9], but the results could not be repeated in ferrets [32]. The group proposed that the HA ministem vaccine candidates need “enhancement” to be effective in large, outbred species (like humans), which motivated us to test their antigen as a noro-VLP conjugate. We executed immunogenicity tests in a similar setting. SpyCatcher-HA2 and HA2-noro-VLP (this study) and HA2 alone [12] all raised high antibody titers, but SpyCatcher-HA2 and HA2-noro-VLP were able to do so without added adjuvant and with 2- or 20-fold smaller antigen doses, respectively. Noro-VLP display showed a trend towards higher antibody titers as compared to SpyCatcher-HA2 and with lower variation. From this indirect comparison, it can be deduced that especially noro-VLP display and even SpyCatcher conjugation alone have adjuvant effects surpassing or at least comparable to CpG7909 (TriLink BioTechnologies, USA), used by Mallajosyula et al. Additionally, bacterial endotoxins were not removed or measured from the *E. coli* produced antigens in the earlier reports, so the adjuvant load might have been high in these immunization tests. Therefore, our HA2 vaccine candidate represents a product closer to clinical use.

In this study, we observed no M2e-specific antibodies even when vaccinating mice with noro-VLP decorated with the M2e peptide. Instead, all antibodies we detected in the mouse sera in ELISA seemed to identify the SpyCatcher conjugation protein. Anti-SpyCatcher antibodies are to be expected, since SpyCatcher is derived from a common human pathogen, *Streptococcus pyogenes* [7]*,* and the immunogenicity of the full-length protein has been noted in previous immunization studies [33]. Nevertheless, we expected to also find M2e-specific antibodies, since it has been shown to be immunogenic with similar platforms [34, 35]. Both papers presented N-terminally fused M2e, whereas we fused M2e to the C-terminus of SpyCatcher (Additional file 1: Table S1). As M2e represents the N-terminus of the influenza M2 protein, this change in topology might be problematic for natural folding, although some protective antibodies have been obtained also from M2e presented in internal loops [36]. Directing immunity against the peptide presented on the noro-VLP could work better with 3–5 tandem repeats of the peptide, as in [36, 37]. Now that we have shown that noro-VLP tolerates C-terminal HisTag [6] and SpyTag fusion well, we should next investigate direct genetic fusion of M2e, removing the immunogenic SpyCatcher from the product. Another way to remove SpyCatcher and still maintain a modular system would be to test the SnoopLigase 3-part conjugation technology with the noro-VLP [38].

M2e and HA stem vaccines both work in a distinct mechanism as compared to conventional influenza vaccines, which makes studying their neutralizing effect more complicated. HA2-stem-directed antibodies mediate neutralization of influenza by inhibiting membrane fusion [12], while M2e antibodies are thought to mainly work against infected cells that express M2e on their membrane [10]. Neither block influenza from binding its host cell receptors, like whole virus vaccines, which means that the convenient hemagglutination assay usually used for estimating neutralization capacity of influenza vaccines will not work in this case. Cell-based or challenge studies with live influenza virus should still be executed to further assess the antibodies we obtained.

## Conclusions

In this study, we constructed a modular vaccine platform based on the noro-VLP. The SpyTagged noro-VLP can be decorated with SpyCatcher fused antigens by simply mixing the components together in a variety of solutions. We established efficient, scalable and easily modifiable production and purification methods for the SpyTag-noro-VLP platform and two prototype antigens. Important for a vaccine platform, our studies demonstrate that the SpyTag-noro-VLP is stable and that it can be stored at normal refrigerator temperature for months. We decorated the noro-VLP with two conserved influenza antigens, aiming for a universal influenza vaccine. In mouse immunization experiments, the decorated noro-VLPs raised high titers of IgG antibodies against HA2 and SpyCatcher proteins. Presentation of HA2 on noro-VLP showed a trend towards higher antibody titers compared to soluble SpyCatcher-HA2 alone. In conclusion, the SpyTag-noro-VLP vaccine platform offers a convenient and promising method to rapidly produce vaccine candidates out of various protein domain antigens, but further research is still needed to confirm compatibility of the noro-VLP platform with peptide antigens.

## Methods

### Construction of expression plasmids

The genes for SpyCatcher-fused influenza M2e or HA2 antigens were codon-optimized for *E. coli* expression and synthesized by GenScript (USA). The sequence of N- and C-terminally truncated, 84-amino-acid-long SpyCatcher [39], was used in the constructs. We included an N-terminal HisTag in SpyCatcher and made it cleavable by including a TEV protease site. The antigens were fused to the C-terminus of SpyCatcher via a two-residue (LE) linker, which makes up an XhoI restriction site on the DNA level and allows for convenient replacement of the antigen. For the ELISA assays, we ordered a plasmid encoding HA2 without the SpyCatcher fusion. GenScript prepared it by restriction-ligation and subcloning of the SpyCatcher-HA2 plasmid. The amino acid sequences of the proteins produced during this study are shown in Additional file 1: Table S1. The genes were subcloned into the pET-11b (+) plasmid under the strong T7 promoter and sequence-verified.

To construct a gene encoding SpyTag-noro-VLP, the DNA sequence of SpyTag was inserted into the C-terminus of VP1 from norovirus strain Hu/GII.4/Sydney/NSW0514/2012/AU (GenBank accession no. AFV08795). SpyTag is separated from VP1 by a 4-residue linker (TSGG), containing a unique SpeI restriction site. GeneArt (Germany) codon-optimized the SpyTag-noro-VLP gene for insect cell expression and synthesized and subcloned it into the pFastBac Dual vector, under the polyhedrin promoter. High-titer baculovirus stock expressing the SpyTag-noro-VLP gene was produced in Sf9 insect cells with the standard Bac-to-Bac protocol (Thermo Fisher Scientific, USA). Baculovirus titers were determined with the BacPAK Baculovirus Rapid Titer Kit (Takara Bio, Japan, #631406).

### Expression and purification of SpyTagged norovirus-like particles

The SpyTag-noro-VLP-expressing baculovirus stock was used to infect Hi5 insect cells at a cell density of 2*10^6^ cells/mL with a multiplicity of infection value of 1. The production medium was collected 4–6 days post infection and clarified by vacuum-filtering through Nalgene Rapid flow 0.2 μm filter (Thermo Fisher Scientific, USA, #566-0020) with the help of SartoClear Dynamics Lab FilterAid (Sartorius, Germany, SDLV-0500-10C-2). The clarified production medium was loaded on 30% sucrose cushions in PBS and centrifuged for 16–22 h in 104,000–175,000*g*. The resulting pellets were dissolved in sterile PBS and the concentrated SpyTag-noro-VLP was diluted in 20 mM phosphate buffer (pH 7.0) until conductivity reached < 5 mS/cm.

Next, the diluted supernatant was loaded on a pre-packed 5 mL HiTrap Q XL anion exchange column (GE Healthcare, USA, 17-5159-01) using a flow rate of 2 mL/min. The flow rate was raised to 3 mL/min for the rest of the chromatography purification. Weakly bound proteins were washed out with 5 column volumes (CV) of binding buffer (50 mM phosphate buffer, pH 7.0). The rest of proteins in the column were eluted by linearly increasing the concentration of elution buffer (binding buffer with 1 M NaCl) over 20 CV. The VLP-containing fractions were pooled, concentrated to 1–2 mg/mL and simultaneously buffer exchanged to PBS (pH 7.2) with VivaSpin Turbo 15 10,000 molecular weight cut-off (MWCO) PES tubes (Sartorius, Germany, #VS15T02). The product was sterile-filtered and stored at + 4 °C until further use.

### Expression and purification of SpyCatcher-fused influenza antigens

After sequential optimization of the most important parameters (strain, induction time point, temperature), BL21 Star (DE3) *E. coli* (Thermo Fisher Scientific, USA, #C601003) expressed the SpyCatcher-M2e fusion protein at + 25 °C overnight. Induction was done with 1 mM IPTG when optical density (OD_600nm_) reached ~ 0.6. The bacteria were pelleted by centrifugation (10 min, 4000 g), resuspended in binding buffer (20 mM PBS, 20 mM imidazole, 500 mM NaCl; pH 7.4) and lysed with Avestin Emulsiflex C3 (ATA Scientific, Australia) homogenizer (2 rounds, 80 bar). The lysates were clarified by centrifugation (20,000*g*, 20 min, + 4 °C) and loaded on an affinity column (5 mL HisTrap FF crude; GE Healthcare, USA, #17528601). Residual endotoxins were washed from the column-bound proteins with 50 CV 0.1% Triton X-114 in binding buffer (as described in [40]), and the target proteins were eluted with imidazole. We optimized SpyCatcher-H1F production likewise. After induction with 1 mM IPTG at OD_600nm_ ≈ 0.8, it was expressed in C41 *E. coli* at + 18 °C overnight and purified identically. Both proteins were dialyzed thrice into PBS with gradually decreasing concentrations of imidazole and EDTA, concentrated with appropriate VivaSpin columns if needed and, finally, flash-frozen for storage at − 80 °C.

### Conjugation of antigens on norovirus-like particles

The purified SpyTag-noro-VLPs were mixed with a twofold molar excess of SpyCatcher-fused influenza antigens and incubated for 2 h at RT or overnight at + 4 °C. To remove unreacted SpyCatcher-antigen, the product was dialyzed four times using Spectra/Por 1000 kDa MWCO membrane (Spectrum laboratories, USA, #131486) in at least 200-fold volume of PBS.

### Characterization of purified proteins and particles

The size and polydispersity of the produced proteins and particles were measured by dynamic light scattering (DLS) with the Zetasizer Nano ZS (Malvern Instruments, UK). Protein purity and conjugation efficiency were estimated by densitometry analysis of Stain-free (Bio-Rad, USA) or silver stained SDS-PAGE gels with the Image Lab software (Bio-Rad, USA). For confirming the identity of the purified influenza antigens, we transferred the proteins from gel onto membrane with the semi-dry Trans-blot Turbo system (Bio-Rad, USA). The affinity tags in the proteins were identified by a mouse monoclonal anti-HisTag antibody (1:5000, Thermo Fisher Scientific, USA, #ma1-21315). The absence of baculovirus in the purified VLPs was confirmed with mouse monoclonal anti-gp64 antibody (1:2000, Santa Cruz Biotechnology, USA, #sc-65499). The bound primary antibodies were visualized by IRDye 800CW goat anti-mouse IgG (1:20,000, LI-COR Biosciences, USA, #926–32210) secondary antibody and the Odyssey CLx instrument (LI-COR Biosciences, USA).

F200 S/TEM (Jeol, Japan) transmission electron microscope (TEM) was used to examine the morphology and size of noro-VLPs after negative staining with 1% uranyl acetate. For size distribution analysis, images of n > 100 particles of each class were measured with Fiji ImageJ software [41] by manually outlining each particle, using the software’s area function and calculating the diameter of an equivalent circle based on the area. Before analysis, the image files and folders were encoded to blind the measurer. Pierce BCA protein assay (Thermo Fisher Scientific, USA, #23252) was used to measure total protein concentrations in preparations. Endotoxin concentrations were determined with Pierce LAL Chromogenic Endotoxin Quantitation Kit (Thermo Fisher Scientific, USA, #88282). The amount of residual DNA was measured with the Quant-iT dsDNA high sensitivity kit (Thermo Fisher Scientific, USA, #Q33120).

The thermal and pH stability of the decorated VLP particles was characterized by differential scanning fluorimetry (DSF), as described in [42]. Briefly, SYPRO Orange (Thermo Fisher Scientific, USA, S6650), a fluorescence dye that binds to hydrophobic amino acid residues, was used to analyze the unfolding or denaturation of the VLPs to study the conformational stability of the vaccines. The fluorescence intensity of the dye in the presence of the vaccines was plotted as a function of the temperature, and melting temperatures (T_m_) of the vaccines were derived from the inflection points of the transition curve using the Boltzmann equation [43]. Before pH stability studies, native and SpyTagged noro-VLP were dialyzed three times (final dialysis overnight) into 20 mM phosphate-citrate buffer, pH 3, 5.5 or 8.

### Immunizations

Specific pathogen-free female BALB/c OlaHsd mice, aged 6 weeks (Envigo, Horst, the Netherlands), were randomly divided into five groups (I–V, 4–5 mice/experimental group) and acclimatized under controlled specific conditions for a week before starting the study. The animals were immunized at study weeks 0 and 3 intramuscularly (i.m.) at the right caudal thigh muscle with SpyTagged noro-VLPs, influenza antigens presented on noro-VLPs or SpyCatcher-fused influenza antigens (Table 1). I.m. injection was chosen to mimic human influenza vaccines currently in use, but at the same time it restricted our maximum dose volume to 50 µL. Our hypothesis at the time was that the SpyCatcher-antigen proteins by themselves would be very poor as immunogens, so we tried rather high doses of them to be able to claim so. The antigen doses indicated in Table 1 were calculated by multiplying total protein dose (measured by BCA) with the proportion of the antigen protein in the preparation, considering the estimated conjugation efficiency and the antigen’s mass, compared to the mass of other proteins in the particle system. For example, HA2-noro-VLP was diluted in PBS to the concentration of 0.3 mg/mL to reach a total protein mass of 15 µg in a dose sized 50 µL. Based on SDS-PAGE densitometry, 51% of all protein in the HA2-noro-VLP preparation was HA2-noro-VP1, while the rest was unconjugated noro-VP1. The molecular masses of noro-VP1, SpyCatcher and HA2 are 61, 15.50 and 15.48 kDa, respectively. Thus, the specific mass of HA2 in the dose can be calculated as follows: $51\%*15 \mu \mathrm{g}*(\frac{15.48 \mathrm{kDa}}{\left(61+15.50+15.48\right)\mathrm{kDa}})=1.3 \mu \mathrm{g}$. No external adjuvants were included in any vaccine formulation. Immunizations were performed under general anesthesia by inhalation of isoflurane (Attane vet, Vet Medic Animal Health Oy, Parola, Finland, #AP/DRUGS/220/96). Whole blood was collected at the time of sacrifice (study week 5) and processed according to the previously published procedure [44].

### ELISA

Sera of individual mice were tested in enzyme-linked immunosorbent assay (ELISA) for the presence IgG antibodies against HA2, Spycatcher-HA2 and Spycatcher-M2e, as described earlier [45]. Briefly, half-area polystyrene 96-well-plates (Corning Inc., #3690) were coated with 50 ng of HA2 or 500 ng of SpyCatcher-fused protein per well. Antigen-specific IgG antibodies in the sera were detected with horseradish peroxidase -conjugated anti-mouse IgG (Sigma-Aldrich, Cat. A4416) and SIGMA FAST OPD substrate (Sigma-Aldrich, Cat. P9187). Optical densities at 490 nm (OD_490_) were measured with a microplate reader (Victor^2^, PerkinElmer, USA). Endpoint titers were defined as the reciprocal of the highest serum dilution with an OD_490_ above the positivity cut-off value (> 0.1 OD_490_ unit). The difference between non-parametric observations in independent vaccine groups was determined with the Mann–Whitney *U* test. For data analysis, we used GraphPad Prism software, version 8.4.2, and defined that *p* < 0.05 indicates statistically significant difference.

## Supplementary Information

**Additional file 1: Figures S1–S5 and Table S1.** The figures provide additional results and graphical presentations from characterization of the used vaccine candidates and their components. The amino acid sequences of the used vaccine components are listed in Table S1.

## Data Availability

The datasets used and/or analyzed during the current study are available from the corresponding author on reasonable request.
