# Supplementary material for: Modular vaccine platform based on the norovirus-like particle
Source: J Nanobiotechnology. 2021 Jan 19;19:25. doi: 10.1186/s12951-021-00772-0 (PMC7815183; doi:10.1186/s12951-021-00772-0)
Supplement: Supplementary file 1 — Additional file 1: Figures S1–S5 and Table S1. The figures provide additional results and graphical presentations from characterization of the used vaccine candidates and their components. The amino acid sequences of the used vaccine components are listed in Table S1. [file 12951_2021_772_MOESM1_ESM.docx]

**Modular vaccine platform based on the norovirus-like particle**

Vili Lampinen^1^, Suvi Heinimäki^2^, Olli H. Laitinen^1^, Marko Pesu^1, 3^, Minna M. Hankaniemi^1*^, Vesna Blazevic^2*^, Vesa P. Hytönen^1,3*#^

*Contributed equally

^1^Faculty of Medicine and Health Technology, Tampere University, FI-33014 Tampere, Finland

^2^Vaccine Development and Immunology/Vaccine Research Center, Faculty of Medicine and Health Technology, Tampere University, Finland

^3^Fimlab Laboratories, Tampere, Finland

## ^#^Corresponding author: Vesa Hytönen, vesa.hytonen@tuni.fi, Faculty of Medicine and Health Technology, Tampere University, FI-33014 Tampere, FinlandFigure S1


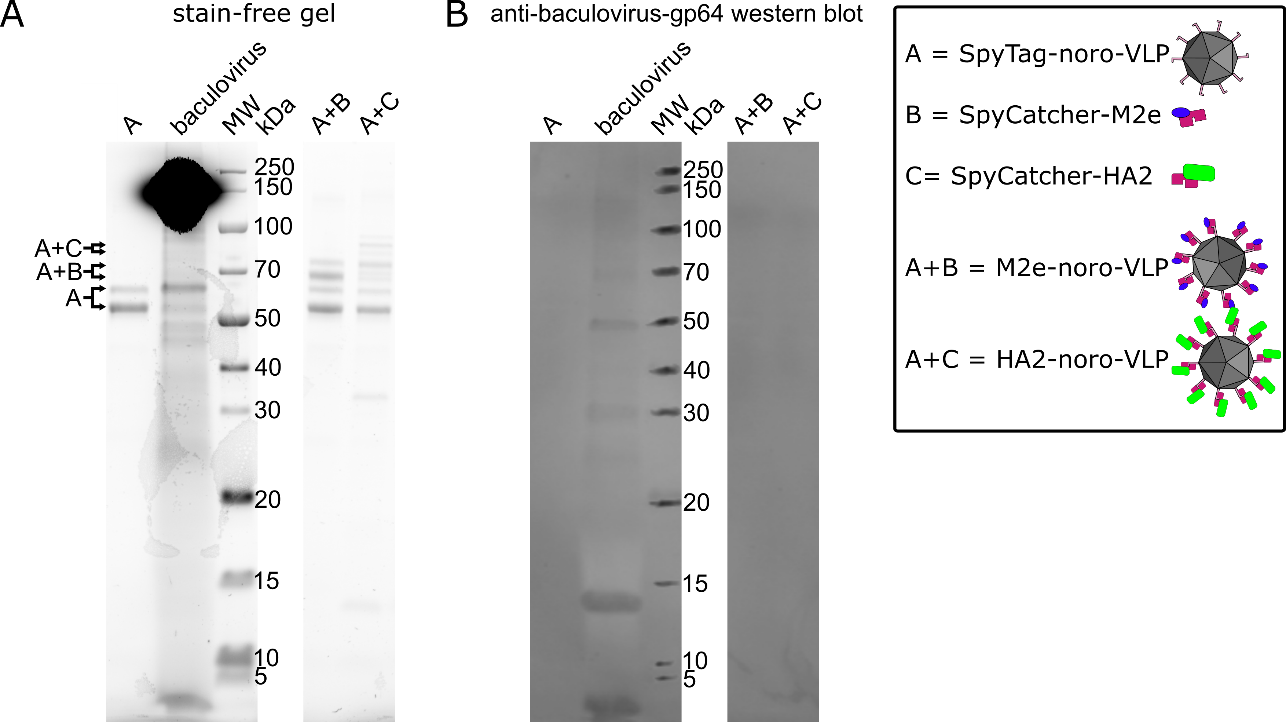


Figure S1. Anti-gp64 western blotting confirms the absence of baculovirus in the purified VLP samples. The vaccine candidates and SpyTag-noro-VLP alone (~1 µg/well) were run on stain-free gel (A) and blotted with anti-baculovirus-gp64 antibody (B). Baculovirus protein was only detected in the positive control well with baculovirus.

## Figure S2


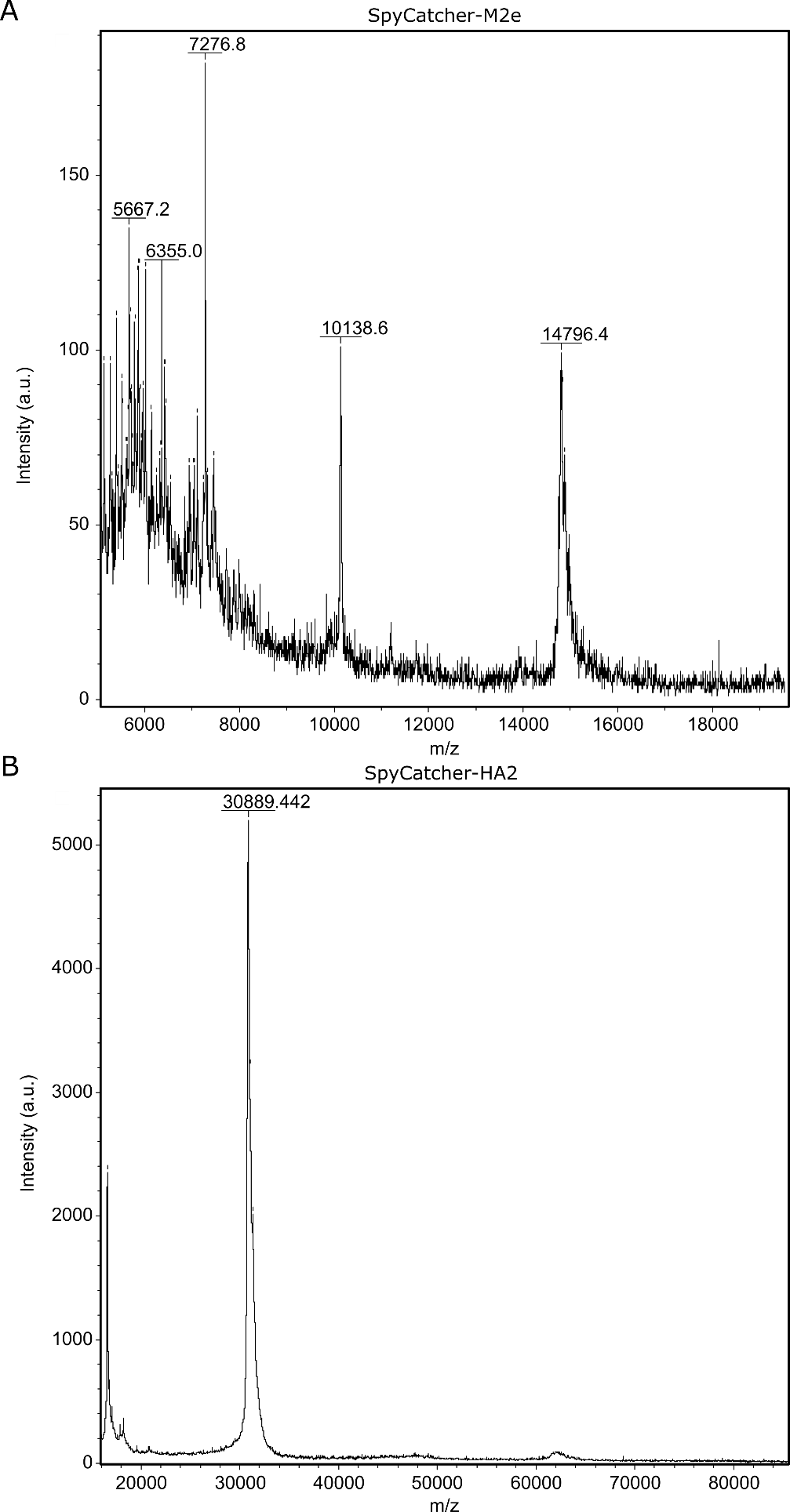


Figure S2. MALDI-MS show that SpyCatcher influenza antigens are produced in their full-length form. The masses of purified SpyCatcher-M2e (A) and SpyCatcher-HA2 (B) were measured with MALDI-MS. The largest peaks correspond to the predicted masses of both proteins, confirming that the bands observed in SDS-PAGE represent the full-length protein.

## Figure S3


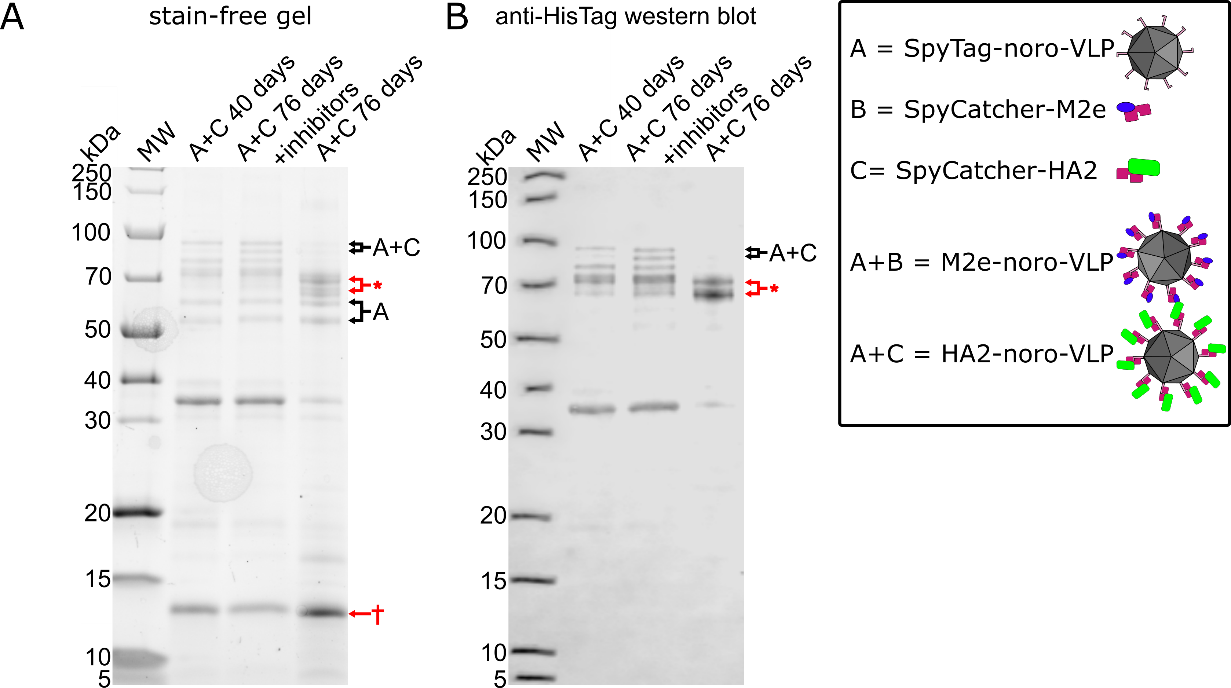


Figure S3. Prolonged storage of conjugated HA2-noro-VLP induces proteolysis. SpyTag-noro-VLP was conjugated with HA2 and then stored at +4 °C for 76 days with or without 1 µg/mL of aprotinin and leupeptin protease inhibitors. The samples were run on Stain-free SDS-PAGE gel (A) and blotted with anti-HisTag antibody (B). Degradation of HA2 makes the largest two bands (labeled A+C) move down (red *). This also increases prominence of an unknown ~13 kDa band (red †), invisible in anti-HisTag western blot.

## Figure S4

Figure S4. Supporting data for DLS measurements. A) Dynamic light scattering analyses executed monthly during a 5-month storage period at +4 °C in pH 7.4 PBS. This data is presented numerically in figure 3A. B) Dynamic light scattering analysis of SpyTag-noro-VLP and SpyTag-noro-VLP decorated with M2e and HA2. The data is presented graphically in figure 2C.


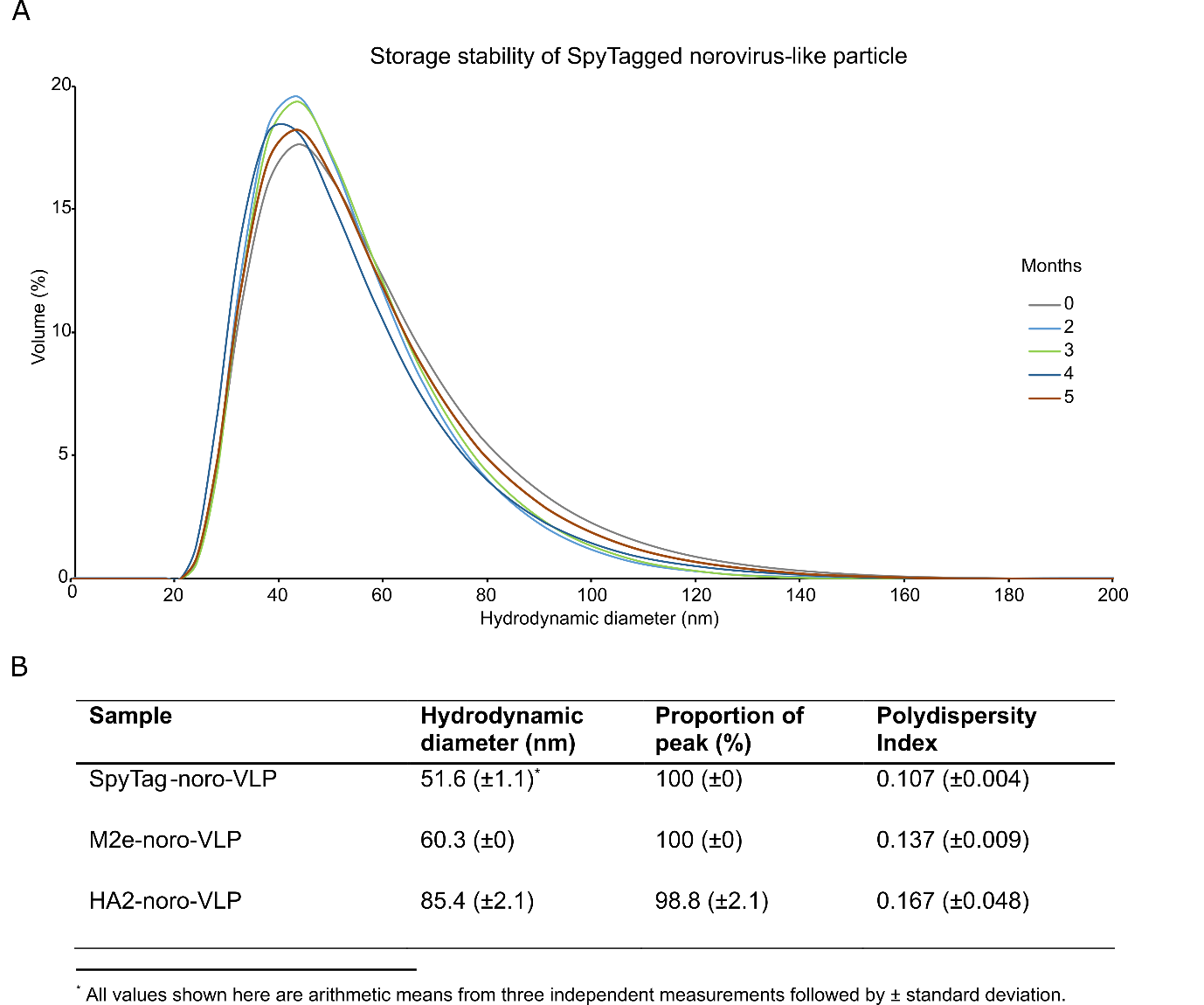


## Figure S5

Figure S5. SpyTag affects the environmental stability of noro-VLP only slightly. Native and SpyTagged noro-VLP samples were dialyzed thrice into 20 mM citrate phosphate, pH 3, 5.5 or 8. After the final dialysis step, the melting temperature (T_m_) of noro-VLP was measured in different pH values with differential scanning fluorimetry (DSF). A) Plotted here are the arithmetic means of normalized fluorescence from three independent measurements.
B) This summary table of mean melting temperatures ± standard deviations includes the control measurement of noro-VLP in pH 7.4 PBS (Figure 3).


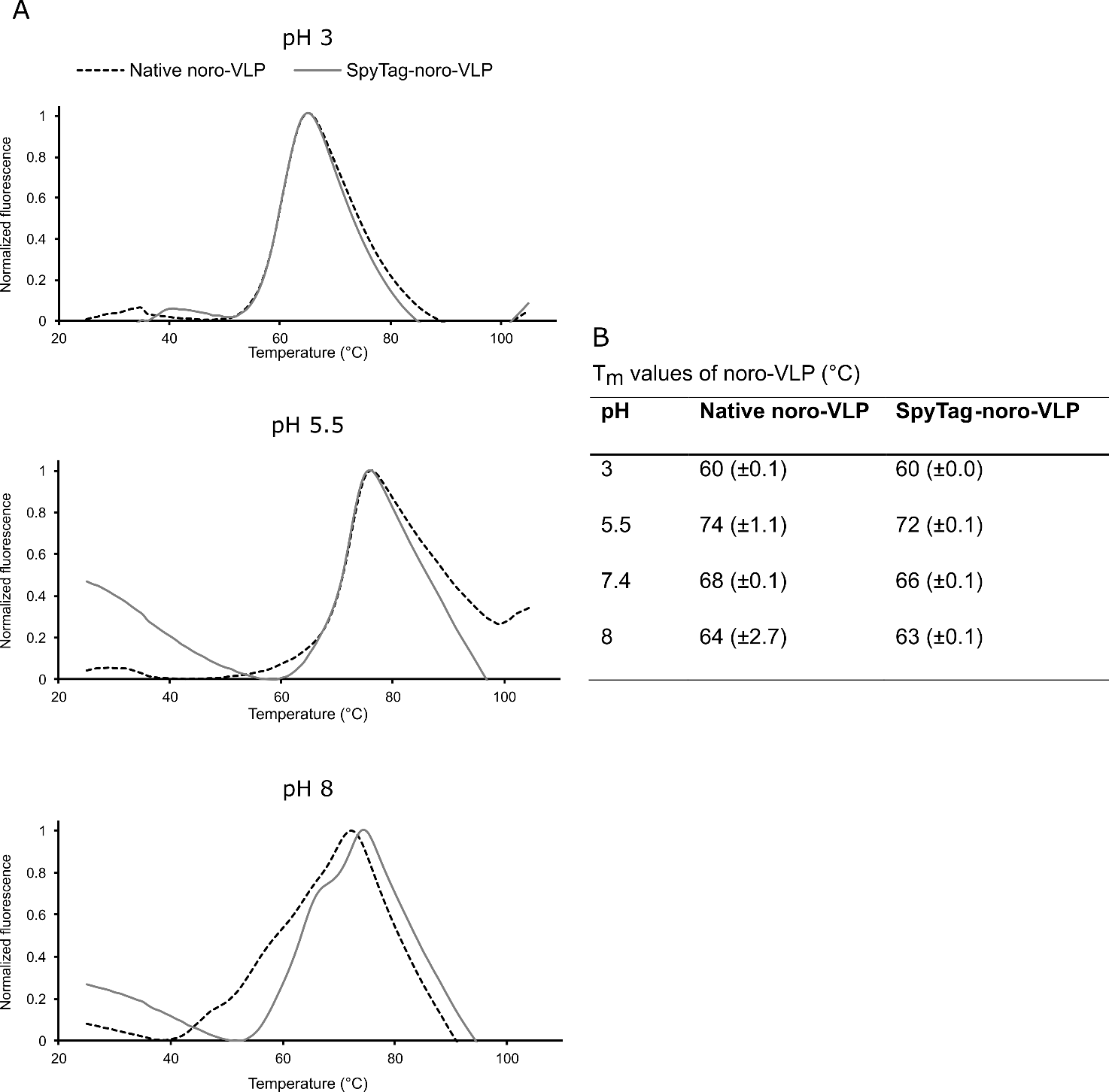


## Table S1

SpyCatcher-M2e (His-tag, TEV-site, SpyCatcher, XhoI, M2e):

MHHHHHHDYD IPTTENLYFQ GSGDSATHIK FSKRDEDGKE LAGATMELRD SSGKTISTWI 60

SDGQVKDFYL YPGKYTFVET AAPDGYEVAT AITFTVNEQG QVTVNGLE**MS LLTEVETPIR** 120

**NEWGCRCNDS SD**** 132

SpyCatcher-HA2 (His-tag, TEV-site, SpyCatcher, XhoI, HA2-Foldon):

MHHHHHHDYD IPTTENLYFQ GSGDSATHIK FSKRDEDGKE LAGATMELRD SSGKTISTWI 60

SDGQVKDFYL YPGKYTFVET AAPDGYEVAT AITFTVNEQG QVTVNGLE**DT VDTVLEKNVT** 120

**VTHSVNLLED SHGSANSSLP YQNTHPTTNG ESPKYVRSAK LRMVTGLRNG SAGSATQNAI** 180

**NGITNKVNTV IEKMNIQDTA TGKEFNKDEK RMENLNKKVD DGFLDIWTYN AELLVLLENE** 240

**RTLDAHDS**QG TGGGYIPEAP RDGQAYVRKD GEWVLLSTFL ** 280

HA2 (His-tag, TEV-site, HA2-Foldon):

MHHHHHHDYD IPTTENLYFQ **DTVDTVLEKN VTVTHSVNLL EDSHGSANSS LPYQNTHPTT** 60

**NGESPKYVRS AKLRMVTGLR NGSAGSATQN AINGITNKVN TVIEKMNIQD** **TATGKEFNKD** 120

**EKRMENLNKK VDDGFLDIWT YNAELLVLLE NERTLDAHDS** QGTGGGYIPE APRDGQAYVR 180

KDGEWVLLST FL** 194

SpyTag-noro-VLP (noro-VP1-SpeI-SpyTag)

MKMASSDANP SDGSAANLVP EVNNEVMALE PVVGAAIAAP VAGQQNVIDP WIRNNFVQAP 60
GGEFTVSPRN APGEILWSAP LGPDLNPYLS HLARMYNGYA GGFEVQVILA GNAFTAGKVI 120
FAAVPPNFPT EGLSPSQVTM FPHIVVDVRQ LEPVLIPLPD VRNNFYHYNQ SNDPTIKLIA 180
MLYTPLRANN AGDDVFTVSC RVLTRPSPDF DFIFLVPPTV ESRTKPFSVP VLTVEEMTNS 240
RFPIPLEKLF TGPSSAFVVQ PQNGRCTTDG VLLGTTQLSP VNICTFRGDV THITGSRNYT 300
MNLASQNWND YDPTEEIPAP LGTPDFVGKI QGVLTQTTRT DGSTRGHKAT VYTGSADFAP 360
KLGRVQFETD TDRDFEANQN TKFTPVGVIQ DGGTTHRNEP QQWVLPSYSG RNTHNVHLAP 420
AVAPTFPGEQ LLFFRSTMPG CSGYPNMDLD CLLPQEWVQY FYQEAAPAQS DVALLRFVNP 480
DTGRVLFECK LHKSGYVTVA HTGQHDLVIP PNGYFRFDSW VNQFYTLAPM GNGTGRRRAV 540
TSGG**AHIVMV DAYKPTK** 557
